# An advanced computational bioheat transfer model for a human body with an embedded systemic circulation

**DOI:** 10.1007/s10237-015-0751-4

**Published:** 2015-12-26

**Authors:** Alberto Coccarelli, Etienne Boileau, Dimitris Parthimos, Perumal Nithiarasu

**Affiliations:** 1 Wales Heart Research Institute, School of Medicine, Cardiff University, Cardiff, UK; 2 Zienkiewicz Centre for Computational Engineering, College of Engineering, Swansea University, Swansea, UK

**Keywords:** Systemic circulation, Bioheat transfer, Heat conduction, Convection, Perfusion, Thermoregulation, Finite element method

## Abstract

In the present work, an elaborate one-dimensional thermofluid model for a human body is presented. By contrast to the existing pure conduction-/perfusion-based models, the proposed methodology couples the arterial fluid dynamics of a human body with a multi-segmental bioheat model of surrounding solid tissues. In the present configuration, arterial flow is included through a network of elastic vessels. More than a dozen solid segments are employed to represent the heat conduction in the surrounding tissues, and each segment is constituted by a multilayered circular cylinder. Such multi-layers allow flexible delineation of the geometry and incorporation of properties of different tissue types. The coupling of solid tissue and fluid models requires subdivision of the arterial circulation into large and small arteries. The heat exchange between tissues and arterial wall occurs by convection in large vessels and by perfusion in small arteries. The core region, including the heart, provides the inlet conditions for the fluid equations. In the proposed model, shivering, sweating, and perfusion changes constitute the basis of the thermoregulatory system. The equations governing flow and heat transfer in the circulatory system are solved using a locally conservative Galerkin approach, and the heat conduction in the surrounding tissues is solved using a standard implicit backward Euler method. To investigate the effectiveness of the proposed model, temperature field evolutions are monitored at different points of the arterial tree and in the surrounding tissue layers. To study the differences due to flow-induced convection effects on thermal balance, the results of the current model are compared against those of the widely used modelling methodologies. The results show that the convection significantly influences the temperature distribution of the solid tissues in the vicinity of the arteries. Thus, the inner convection has a more predominant role in the human body heat balance than previously thought. To demonstrate its capabilities, the proposed new model is used to study different scenarios, including thermoregulation inactivity and variation in surrounding atmospheric conditions.

## Introduction

A fundamental understanding of heat transport in a human body is important for studying a broad range of situations, such as the effect of temperature controlled surgeries and the impact of dramatic change in atmospheric or surrounding temperature. The thermal manipulation of body temperature has been used for a long time in cryosurgery and cancer treatment (hyperthermia) (Wells et al. 2005; Zohdi 2014; Zhu et al. 2005). It is also known that dramatic changes in weather (extremely cold seasons or heat waves) can lead to adverse health conditions and in some cases death. In thermally controlled surgeries, understanding the heat or cold dissipation mechanism in a human body can be critically important. In a more ordinary situation, seasonal changes in atmospheric temperature require the body to adapt fast to keep the stimuli under control. Despite all the regulatory mechanisms of the human body, whenever temperature reaches a threshold value, tissue damage and/or alterations to biological processes may occur. When subjected to varying temperature conditions, the thermoregulatory system enables the control mechanisms of the body to keep tissue temperature within the threshold limit. Modelling such a control mechanism and resulting temperature behaviour in time and space within a human body is extremely complex. A number of modelling attempts have been made in the past using lumped models or models that have only accounted for conduction and perfusion to understand the heat transfer in a human body. While these models provide a good starting point, they are not comprehensive as they do not include the effect of pulsatility and flow in arteries. A brief overview of the existing models are given below.

The available bioheat transfer models for a human body may be conveniently divided into lumped models, segmented models, and multi-dimensional models. In addition, there are models that fall between these categories. The lumped models are the simplest as they treat the human body as a single point with temperature change allowed only in time. Since the thermoregulation effects and heat transfer processes within the body were not accounted in the lumped models, application of such models are extremely limited. By comparison, fully three-dimensional models represent thermal changes in a human body close to reality. Such representations are, however, complex, extremely expensive, and difficult to employ. Thus, the segmented models that carefully account for various biological and physical processes are probably the best models for understanding the human body bioheat transfer.

Alternatives to lumped models were developed in the late sixties, and the first of such models consists of two nodes, representing a human body using two concentric shells. The first central shell represents internal organs, bone, muscle, and subcutaneous tissue, and the outer second shell represents the skin layer. The model presented by Gagge et al. (1967) is one of the best-known two-node models. It calculates the thermal response by means of two energy balance equations, one for the core node and one for the skin node. Gagge et al.’s model accounts for the effects of heat accumulation, conductive and convective heat transfer via blood flow between the core and skin shells, and metabolic heat generated during exercise and shivering. The energy exchange with environment has been modelled by considering respiration, convection, radiation, and evaporation of moisture. Gagge et al.’s model is simple to use, but it can only be applied to situations with moderate levels of activity and uniform environmental conditions. Although this model is an improvement of lumped models, it does not allow for the computation of detailed body temperature distribution.

An obvious extension to the two-node model is to introduce multi-nodes to discretely represent different parts of the body.  Stolwijk (1971, 1977), Stolwijk and Hardy (1966) divided the human body into five cylindrical parts to individually represent the trunk, arms, hands, legs and feet, and a spherical body part for the head. Each part was further divided into four concentric shells representing the core, muscle, fat, and skin layers. In this model, the blood circulation system is represented by a blood pool located in the trunk. The trunk is connected to all tissue nodes by a network of blood vessels. The heat transfer between tissue nodes occur by conduction, while between the central blood node and the adjacent tissue nodes by convection. The energy balance equation includes heat accumulation, blood convection, tissue conduction, metabolic activity, respiration, and heat transfer to the environment by convection, radiation and evaporation. A thermal control system is also included. A fundamental limitation of this methodology is that it is restricted to isothermal blood flow.

The model developed by Wissler (1964, 1985), consists of six (later 15) elements connected by a vascular system. The vascular network is composed of arteries, veins, and capillaries and the blood temperatures are assumed to be uniform. Arteries connect the heart to the arterial pool of each element and further into the capillaries. From the capillaries, the blood circulates to the venous pool of the element and back to the heart. Between large arteries and large veins countercurrent heat exchange is modelled. It also accounts for breathing losses and is able to simulate transient states. The 3D transient multi-element model developed by Smith (1991) is based on a more realistic representation of the entire human body than previous models. The body here is composed of 15 elements, which are connected by the central macrocirculation and superficial veins. For the blood network, 1D steady-state Newtonian flow is assumed. An accurate evaluation of breathing losses is proposed by considering the respiration cycle. In this thermoregulatory system proposed, variation of skin blood vessel radii during vasomotor response, the sweat rate and the shivering metabolic rate are functions of the core and mean skin temperatures. The 3D approach makes the model suitable for situations with high-temperature gradients or highly non-uniform thermal conditions. However in such model fat and skin layers are modelled as a single layer; this may affect the heat convection to the skin surface carried by blood flow and thus the thermal response of entire body. Moreover, blood perfusion occurring in capillary beds is not considered.

Another relevant work is that of Fiala (1999), Fiala et al. (2001), who divide the body into cylindrical and spherical elements. Such subdivision was enforced whenever a significant change of body tissue properties occurred. The heat produced within the body is dispersed to the environment by convection, radiation, and moisture evaporation at the skin, and in the lungs/respiratory tract. Such multi-layered model consists of annular concentric tissue layers and uses seven different tissue materials: brain, lung, bone, muscle, viscera, fat, and skin. For the solid conduction problem 1D Penne’s equation was used. Body elements are supplied with warm blood from the central pool by the major arteries. Along the pathway, arterial blood exchanges energy with returning veins as in a countercurrent heat exchanger. This was introduced in order to obtain a more realistic distribution of the arterial blood temperature instead of assuming a constant arterial blood temperature for all body elements equal to the temperature of the central blood pool. Perfusion is used as the mechanism of exchange between blood and tissues. The effects of the thermoregulatory system are also accounted. Cropper et al. (2008) coupled the human model of Fiala (1999) with a CFD for external airflow and obtained a tool able to predict the response of body temperature to several detailed local environmental conditions. Tanabe et al. (2002) modelled human thermal system in a similar way. In this case, each individual body elements consists of a core layer and a skin layer and in the centre of each core layer there are artery and vein blood pools. Between the artery and the superficial vein blood pools, an additional vessel is introduced to account for changes in blood flow due to changes in the ambient environment.

The model developed by Huizenga et al. (2001) is based on the Stolwijk model Stolwijk (1971, 1977), Stolwijk and Hardy (1966) as well as on work by Tanabe et al. (2002), but includes several significant improvements, as it can simulate an arbitrary number of human body segments. Each of these segments consists of four body layers (core, muscle, fat, and skin tissues) while a clothing node has been added to model heat and moisture capacitances. The improved blood flow model includes central artery/vein countercurrent heat exchange and blood perfusion model to estimate blood flow to local tissue. The model also calculates the heat transfer by conduction to surfaces in contact with the body. A better estimation of the convection and radiation transfer coefficients, an explicit radiation heat transfer calculation using angle factors and the addition of a radiation heat flux model are notable additions. Besides this, the model allows simulation of any sequential combination of environmental, clothing, and metabolic conditions. Although the latter models with arterial systems  (Fiala 1999; Fiala et al. 2001; Tanabe et al. 2002; Huizenga et al. 2001) represent a step forward, they do not explicitly include a systemic circulation and the resulting inner convection occurring between the arterial blood and wall.

Other notable models include the one developed by The National Renewable Energy Laboratory (NREL)  (Rugh et al. 2004), which contains a detailed simulation of human internal thermal physiological systems and thermoregulatory responses. Another multi-segmented human thermal model developed by Salloum et al. (2007) for bare human body consists of a comprehensive blood network. Flow rates are based on exact physiological data, real dimensioning, and anatomic positions of the arteries in the body. Holopainen (2012) combined the human thermal modelling with a thermal sensation and comfort model inside a simulated building. The models by Karaki et al. (2013) and Rida et al. (2014) also incorporate dynamic thermal response associated with arterio-venous anastomoses (AVA) functions.

Several works including  Daanen (1991), Koscheyev et al. (1998), and Vanggaard et al. report that AVAs in the distal parts of the extremities play a significant role in the heat exchange with the environment. For example, exposure to extremely cold environment causing cold induced vasodilation (CIVD) to protect hands or feet from cold injury and very high-temperature environments causing heat-induced vasoconstriction (HIVC) so that the warm blood cannot reach the human core easily. However, as reported in Daanen (1991), these two exceptions for the AVA function during CIVD and HIVC do not apply to the vasoconstriction or vasodilation of the arterial system triggered by decreased or increased body core temperature.

The ability to appropriately characterize the inner convection between tissue and vessels, introduced here, has been a weak point in many recent models. Following the work by Smith (1991), Sun (2012) derived a comprehensive 3D model that is able to highlight the heat transfer for walking conditions. Although a blood network was included within solid tissues, this was under the assumption that blood nodes exchange heat with surrounding tissue only via conduction. Moreover, vessels were considered inelastic and thus pulsatile velocity was not accounted for. Ferreira and Yanagihara (2009) proposed a 3D conduction model, where arterial and venous flows are considered as reservoirs and the tissue temperature does not account for any flow temperatures. In subsequent work Ferreira and Yanagihara (2012) modelled heat transfer at a steady state in the upper limbs. Although tissue matter was modelled via partial differential equations, the conditions considered were stationary. Furthermore only two different tissues were used, while a reduced, arterial network was adopted.

In the literature, there is no clear evidence on the role of the venous system on the global thermal balance of the body. Indeed the importance of the heat exchanged between arteries and veins is still a matter of debate, as highlighted by an analytical model by Mitchell and Myers (1968), demonstrating no significant countercurrent effect in the human arm. This can be mainly justified because the distance between large arterial and venous vessels is significant; the high-velocity blood flow and the too short length of the vessels may affect further the countercurrent heat exchange. Vanggaard (1975) confirmed that countercurrent heat exchange is of minor importance in total heat exchange. He concluded that countercurrent heat exchange either had to be always 100 % effective or negligible, and naturally opted for the latter.

Some studies have reported heat transfer in the blood but without the surrounding body tissues. For example, Craciunescu and Clegg (2001) analysed the effect of a pulsating blood velocity field on temperature. In their studies they obtained some important results on the relationship between the pulsating axial velocity and temperature profile and the effect of the Womersley number variation. In the work proposed by  Bommadevara and Zhu (2002) a sophisticated bioheat transfer model representing neck is presented. They evaluate temperatures along common and internal carotid arteries for various environmental conditions. However, blood vessels in this study are treated as rigid tubes, and thus the effects of area variations are not accounted for. Ying et al. (2004) proposed a thermofluid model valid for a circulation system of the upper limb which involves arteries, capillaries, and veins. Here, the temperature is evaluated along the network by considering the effects of blood flow rate, transmural pressure, cross-sectional area, and elasticity. However, this model is not comprehensive as reflections due to variations in vessel topology and properties are not accounted. To enhance the accuracy of heat transfer predictions in flexible tubes and tube networks, a robust method has been proposed in Coccarelli and Nithiarasu (2015).

It is obvious from the reported studies that a step change in modelling approach is needed to more effectively address the human body bioheat transfer. It is also necessary to produce a new generation model that forms the basis for future development. In this regard, the present study combines a state-of-the-art systemic circulation model with heat transfer to a segmentation model for body tissue. The present model has been conceived as a combination of a multi-segmental solid model, derived mostly from Fiala et al. (2001) and the arterial modelling methodology proposed by Mynard and Nithiarasu (2008). For the thermoregulatory equations, we refer to Smith (1991). Coupling these components in a single model represents a novel approach to bioheat transfer studies, as it allows for computing the temperature simultaneously in each system, following a methodology recently reported by Coccarelli and Nithiarasu (2015). The blood flow is considered laminar, and a nonlinear wall law is used for describing blood-wall interaction. With these assumptions, the final expressions of governing equations depend only on cross-sectional area, velocity, and temperature of blood. The physical model of the arterial system is adopted from Low et al. (2012), where the flow and pressure distributions in the arterial system are extensively compared to measurement data. The methods for dealing with ventricle, valve, bifurcations, coronary arteries, and peripheral boundaries are detailed in Mynard and Nithiarasu (2008), Boileau et al. (2015) and not discussed in detail in the present work.

As the present methodology is based on two robust models, it is able to respond to a wide spectrum of conditions without losing integrity of the solution. An example to this effect is the straightforward calculation of conduction in the solid system. The inherent robustness of the proposed model is one of its main advantages when compared to relevant recent works such as Salloum et al. (2007) and Karaki et al. (2013). It would be fair to say that these modelling works have demonstrated good performance in simulating various situations, such as the evaluation of local thermal comfort and human physiological responses to cold water immersion. By comparison, the main aim of the proposed work is not to provide an analysis of the thermal performance of a specific subsystem, but to characterize the heat exchanges occurring within the multilayer solid tissues. A further aim is to investigate how the two intrinsically coupled subsystems interact whenever the body is exposed to various external conditions (especially during non-thermal neutral settings). We were thus able to demonstrate that, depending on the conditions, flow may have either a rewarming or cooling effects on the surrounding tissues. These results have emphasized the modulatory role of arterial inner convection. With regard to important processes involving CIVD and AVA, we are aware they would increase the prediction quality of our model. However for the target we aim, accounting for these processes would involve to disproportionately complicate the model. Indeed the present methodology was not developed just to improve on the accuracy of existing models, but rather to provide a robust tool that can probe temperature distribution along tissues and thus provide a different perspective in the study of bioheat transfer within the human body.

In the work presented here, the systemic blood circulation is embedded into a human body model. The human body model consists of multiple segments of solid cylinders representing head, neck, shoulders, thorax, abdomen, thighs, legs, arms, and forearms. The interface between fluid and tissue systems is explained below. The large arteries exchange heat with tissues only by convection and are subdivided into central vessels and transversal arteries. Each artery belongs to one or more predetermined segments. The central arteries are placed along a cylindrical segment axis, while transversal ones pass through the cylinder transversely in multiple directions. The energy exchange in and around small arteries in tissues is accounted for via perfusion. Each tissue layer of a segment is characterized by specific metabolism and perfusion rates that are adjusted by the thermoregulation system.

The numerical scheme used for solving the set of flow and temperature equations is the explicit form of locally conservative Taylor–Galerkin method (LCTG) (Nithiarasu 2004; Thomas and Nithiarasu 2008; Thomas et al. 2008). The conduction problem in the solid part along the radial direction is solved using an implicit finite difference method. Simulations are carried out on a bare human body.

## Equations, computational method, and physical model

### Governing equations

The variables considered in the system are cross-sectional area (*A*), the average values of velocity (*u*) and temperature (*T*) over the artery cross section. Fluid pressure (*p*) is linked to area via the following nonlinear relation (Formaggia et al. 1999; Olufsen et al. 2000).

1 $$\begin{aligned} p= p_\mathrm{ext}+\beta (\sqrt{A}-\sqrt{A_0}) \end{aligned}$$

where $p_\mathrm{ext}$ is the external pressure acting on the walls of the tube, $A_0$ is the unstressed cross-sectional area, and $\beta$ is a characteristic property of elastic material, given as

2 $$\begin{aligned} \beta = \frac{\sqrt{\pi } h E}{A_0 (1-\sigma ^2)} \end{aligned}$$

here *h* is the artery wall thickness, *E* is the Young’s modulus of arterial wall, and $\sigma$ is the Poisson’s ratio.

In the flow model employed, the density ($\rho$) and viscosity ($\mu$) are assumed to be constant. The thermal properties, specific heat ($c_p$), and thermal conductivity (*k*) of the blood are also assumed to be constant. Following some relevant works (Mynard and Nithiarasu 2008; Ying et al. 2004; Coccarelli and Nithiarasu 2015; Sherwin et al. 2003), the conservation laws for a flow in elastic vessels can be written in the following compact form:

3 $$\begin{aligned} \frac{\partial {\bar{\mathbf{U}}}}{\partial t}+\mathbf{H}\frac{\partial {\bar{\mathbf{U}}}}{\partial x} +\frac{\partial {\bar{\mathbf{G}}}}{\partial x}= {\bar{\mathbf{S}}} \end{aligned}$$

with:

4 $$\begin{aligned} {\bar{\mathbf{U}}}= & {} \begin{bmatrix} A \\ u\\ T \end{bmatrix};\quad \mathbf{H}=\begin{bmatrix} u&A&0 \\ {\frac{\beta }{2\rho \sqrt{A}}}&u&0\\ 0&0&u\end{bmatrix}; \nonumber \\ {\bar{\mathbf{G}}}= & {} \begin{bmatrix} 0 \\ 0\\ - \alpha \frac{\partial T}{\partial x}\end{bmatrix} \quad {\text {and}}\quad {\bar{\mathbf{S}}}= \begin{bmatrix}0 \\ -{8\pi \mu \over {\rho }} \frac{u}{A} \\ \left( \frac{2 h_\mathrm{con,in}}{\rho c_p \sqrt{A/\pi }}\right) (T_t-T) \end{bmatrix} \end{aligned}$$

where ${\bar{\mathbf{U}}}$, ${\bar{\mathbf{G}}}$ and ${\bar{\mathbf{S}}}$ are the vectors of primitive variables, the diffusive and source terms, respectively, and $\mathbf{H}$ is the Jacobian matrix. In order to assign boundary conditions and to apply the Taylor–Galerkin method, it is convenient to write the whole system in a linearized de-coupled form. If diffusion and sources are considered negligible ($\frac{\partial {\bar{G}}}{\partial x}=0$ and $\bar{S}=0$ ), the characteristic variables of Eq. (3) may be obtained as in Mynard and Nithiarasu (2008), Coccarelli and Nithiarasu (2015), Formaggia et al. (1999), Sherwin et al. (2003)

5 $$\begin{aligned} w_1 = u + 4 \sqrt{\frac{\beta \sqrt{A}}{2\rho }};~~~ w_2 = u - 4\sqrt{\frac{\beta \sqrt{A}}{2\rho }};~~~ w_3 =T \end{aligned}$$

Rearranging the expressions in Eq. (5), it is possible to express *A* and *u* by means of characteristic variables as

6 $$\begin{aligned} A=\frac{(w_1-w_2)^4}{1024}\left( \frac{\rho }{\beta } \right) ^2;~~~ u=\frac{1}{2}(w_1+w_2) \end{aligned}$$

These relationships are employed at the boundaries to apply boundary conditions.

In the current model a local thermal equilibrium between the venous blood and the tissue temperatures is assumed. The heat transfer by perfusion is assumed to be proportional to the temperature difference between arterial blood entering the tissue and the tissue. In order to evaluate tissue temperature ($T_t$), the one-dimensional bioheat transfer equation in cylindrical coordinates is solved. For a single layer, the heat conduction is described with the following expression (Pennes 1948)

7 $$\begin{aligned} \rho _t c_t \frac{\partial T_t}{\partial t} - k_t \frac{1}{r} \frac{\partial }{\partial r}\left( r \frac{\partial T_t}{\partial r}\right) =q_m +\phi \rho c_p(T-T_t) \end{aligned}$$

In the above equation, *r* is the radial coordinate, $q_m$ is the volumetric heat generation associated with the metabolism, and $\rho _t$, $c_t$, $k_t$, $\phi$ are the density, specific heat, thermal conductivity, and perfusion coefficient of the tissue, respectively. The metabolism term $q_m$ represents mainly the energy generation due to biological processes. If the body is subjected to work, an enhancement of metabolism in muscle tissue occurs.

### Numerical schemes

In this section, a brief overview on the numerical method is provided. Equation (3) requires a scheme with a stabilization term to obtain a stable solution. Thus, in this study the locally conservative Taylor Galerkin (LCTG) method is used (Nithiarasu 2004; Thomas et al. 2008). Applying the LCTG method to mass and momentum equations, it is possible to obtain (Mynard and Nithiarasu 2008)

8 $$\begin{aligned}{}[M_e] \{ \varDelta U \}^{n+1}=\varDelta t\left( [K_e] \{F \}^n + [L_e] \{S \}^n+{f_{\varGamma _{e}}}^n\right) \end{aligned}$$

where $$[M_e]$$, $$[K_e]$$ and $$ [L_e]$$ are the mass matrix, coefficient matrices for convection, Taylor–Galerkin, and source terms, respectively. Each of these are $$2\times 2$$ matrices (for each equation), and this system of equations is solved on individual elements, independent of surrounding elements. Information is transmitted between elements via the numerical flux term that is imposed, for each element, on the boundary (Mynard and Nithiarasu 2008). The $$2\times 2$$ matrices can be evaluated and inverted at the preprocessing stage, which removes the need for any matrix inversions.

Applying LCTG method to the energy equation gives (Coccarelli and Nithiarasu 2015)

9 $$\begin{aligned}{}[M_e]\{ \varDelta T \}^{n+1}= & {} \varDelta t\{ ([K_{eT}] + [D_{eT}]) \{T \}^n\nonumber \\&+\, [L_{eT}] \{T - T_t \}^n + q_{\varGamma _{e}}^n\} \end{aligned}$$

where the matrix $[D_{eT}]$ is the coefficient matrix for diffusion and $q_{\varGamma _{e}}$ is the numerical conduction flux exchanged between two adjacent elements (Nithiarasu 2004).

The time step restrictions for the fluid solver may be computed using the condition (Mynard and Nithiarasu 2008):

10 $$\begin{aligned} \varDelta t= 0.9 \frac{\varDelta x_\mathrm{min}}{c_\mathrm{max}}. \end{aligned}$$

where $\varDelta x_\mathrm{min}$ is the minimum element length and $c_\mathrm{max}$ is the maximum intrinsic wave speed. For the problem of heat conduction through the surrounding tissue, the forward Euler method is used. At the interface between two layers, continuity of flux is imposed. Since the matrix of the linear system is tridiagonal, Thomas algorithm is used to solve the system.

### Physical model architecture and boundary conditions

The human body bioheat transfer may be modelled using a combination of “passive” and “active” systems. The passive part consists of transport in arteries and solid tissues, and the active system is the thermoregulatory part of the model that attempts to keep the body temperature within a predetermined threshold.

**Fig. 1:**
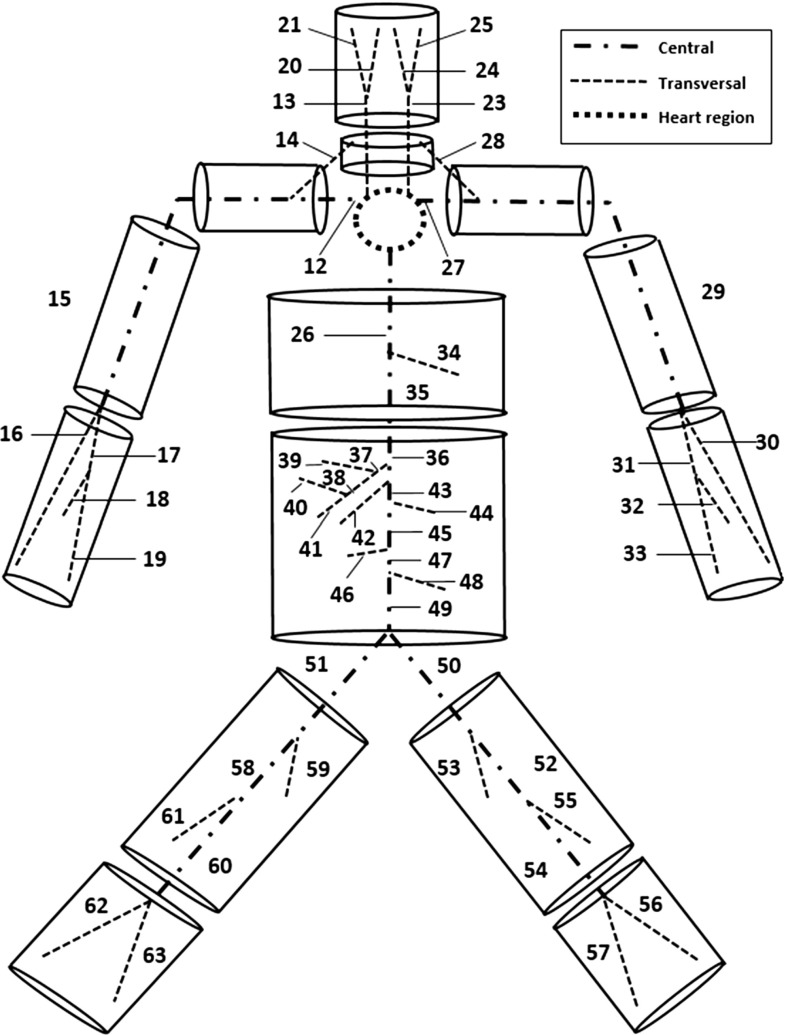
Arterial network considered

#### Arterial network

The systemic circulation is subdivided into large and small vessels. The large arteries are shown in Fig. 1 as proposed in Low et al. (2012). In the present study, only major arteries are included. The micro-circulation is represented by tapering vessels at the extremities of the network (Mynard and Nithiarasu 2008) and the energy exchange occur only through perfusion mechanism. The venous system is not included for the reasons mentioned previously. The whole network is composed by 91 segments (28 are tapering vessels), 6288 elements, and 6379 nodes. Full details about the parameters and dimensions of the network are reported in Low et al. (2012).

**Table 1:** Tissue distribution within body

Cylinder	Tissues	Layer radii (cm)	Length (cm)
Head	Brain, bone, fat, skin	6.6, 7.6, 7.8, 8.0	23.5
Neck	Bone, muscle, fat, skin	1.9, 5.4, 5.6, 5.8	7.9
Thorax	Lung, bone, muscle, fat, skin	7.7, 8.9, 12.3, 12.6 12.9	15.6
Abdomen	Viscera, bone, muscle, fat, skin	7.9, 8.3, 10.9, 12.4, 12.6	24.8
Shoulder	Bone, muscle, fat, skin	3.7, 3.9, 4.4, 4.6	13.4
Arm	Bone, muscle, fat, skin	1.5, 3.4, 4.0, 4.2	29.6
Forearm	Bone, muscle, fat, skin	1.5, 3.4, 4.0, 4.2	23.7
Thigh	Bone, muscle, fat, skin	2.2, 4.8, 5.3, 5.5	58.5
Leg	Bone, muscle, fat, skin	2.2, 4.8, 5.3, 5.5	34.3

Note that the thorax length is smaller than the real average size as heart region is not included

#### Inlet flow conditions

Modelling heart’s pumping action is implemented using the method proposed in Mynard and Nithiarasu (2008), Low et al. (2012). The action of the heart allows one to set inlet boundary conditions and the system includes the left ventricle (LV) and aortic valve (AV) models. The LV is treated as a prescribed forward pressure source, which describes the cardiac cycle and the number of heart beats per unit time or heart rate (HR). The input of the model is a ventricular (forward) pressure prescribed in the ventricle’s point just before the valve. Prescribing inlet and outlet variables is carried out by means of characteristic variables. Rearranging formulations in Eqs. (1) and (5) and by prescribing forward pressure ($p_\mathrm{in}$), it is possible to evaluate the forward characteristic at the inlet ($w_{1\mathrm{in}}$):

11 $$\begin{aligned} w_{1\mathrm{in}}^{n+1}=w_2^0+4 \sqrt{\frac{2}{\rho }}\sqrt{\left( p_\mathrm{in} ^{n+1}-p_\mathrm{ext}\right) +\beta \sqrt{A_0}} \end{aligned}$$

where $w_2^0$ is the initial value of $w_2$ and is also equal to the value of $w_2$ at any time if no backward-running waves reach the inlet. The backward characteristic variable ($w_2$) may be evaluated via linear extrapolation in the $x -t$ plane, where for the next time step $n+1$,

12 $$\begin{aligned} w_2^{n+1}|_{x=x_0}=w_2^{n}|_{x=x_0-\lambda _2^n \varDelta t} \end{aligned}$$

Primitive variables *A* and *u* at the inlet node can be evaluated by using Eq. (6). The behaviour of the AV is represented by a time-varying transmitter and reflector at the inlet. For each impedance of the network, a characteristic reflection coefficient ($R_{z}$) could be defined as

13 $$\begin{aligned} R_{z}=-\frac{\varDelta w_2}{\varDelta w_1}=\frac{w_2^{n+1}-w_2^{0}}{w_1^{n+1}-w_1^{0}} \end{aligned}$$

where $w_1^0$ is the initial value (corresponding to no-pulse situation). Including the contribution from the AV, the total forward characteristic variable ($w_{1\mathrm{in}}^*$) can be written as

14 $$\begin{aligned} w_{1\mathrm{in}}^*=w_{1p} + w_{1r}+w_1^0 \end{aligned}$$

where $w_{1p}$ is the change in the incoming characteristic associated with the ventricular pump and $w_{1r}$ is the change associated with backward-travelling waves that are partially or completely reflected from the valve.

Using Eq. (13) to model the AV impedance, it is possible to write:

15 $$\begin{aligned} w_{1r}=R_{Vr}(t) \varDelta w_2 \end{aligned}$$

where $$R_{Vr}(t)$$ is a time-varying valve reflection coefficient for backward-travelling waves. It is assumed that $$R_{Vr}=0$$ when the valve is open, $$ R_{Vr}=1$$ when it is closed, and that this value varies exponentially when the valve is opening or closing. Further details on the boundary conditions may be obtained from  Mynard and Nithiarasu (2008), Low et al. (2012).

**Fig. 2:**
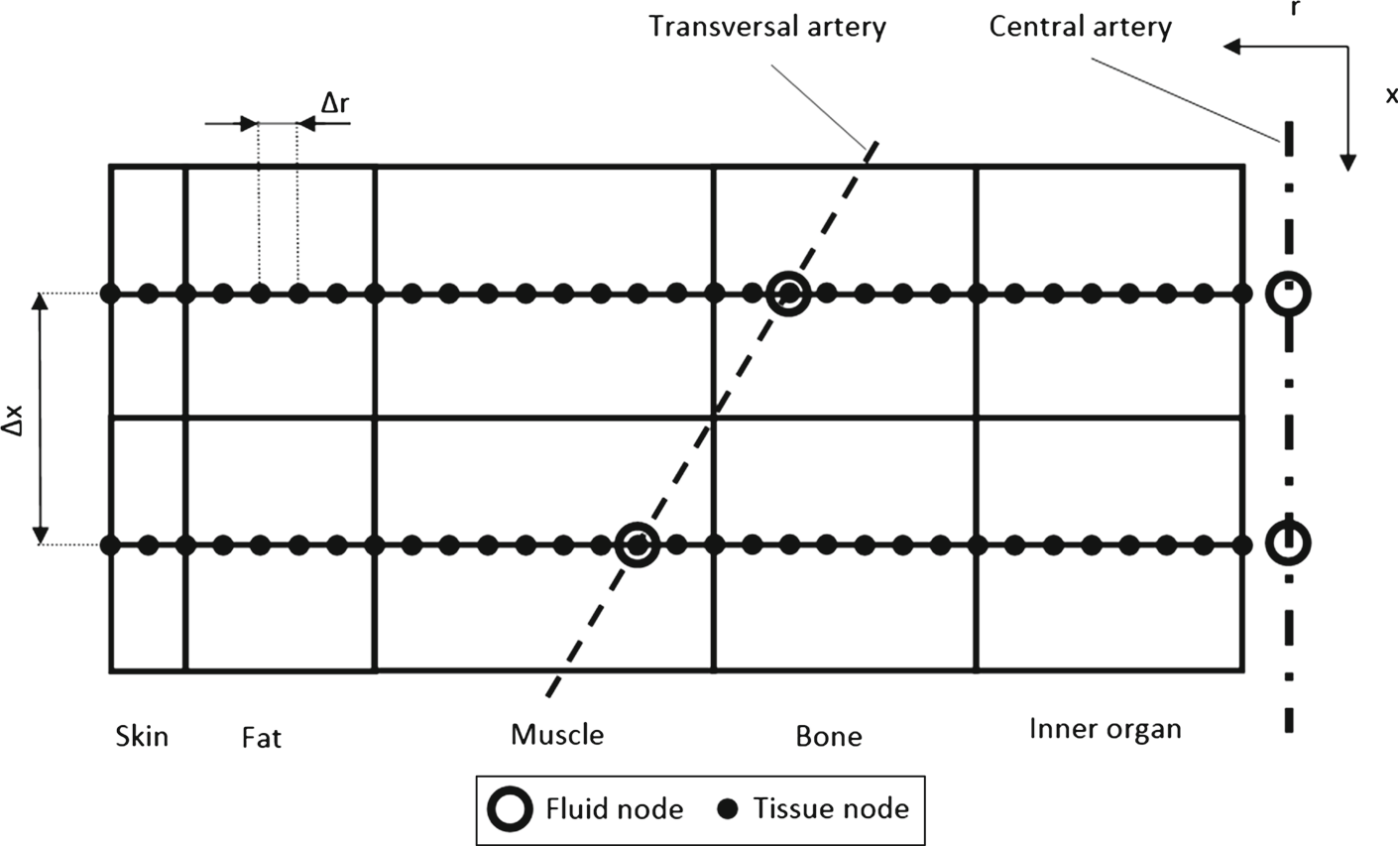
Longitudinal and radial discretizations for one cylinder

#### Extremities and branching points

To model branch ending, tapering vessels are used. These terminal tubes present multiple step decreases in $A_0$ or step increases in $\beta$. Thus characteristic reflections of the downstream vasculature are accounted for. Calculation of the backward characteristic variable on the exit node may also be performed by prescribing reflections at the exit. The reflection coefficient of the terminal vessel ($R_{t}$) can be determined again by means of Eq. (13), while the value of $w_1$ for the next time step $(t =n+1)$ is extrapolated. Thus the unknown $(w_2^{n+1})$ is

16 $$\begin{aligned} w_2^{n+1}=w_2^0-R_{t}\left( w_1^{n+1}-w_1^0\right) \end{aligned}$$

In the present work, $R_{t}$ is set equal to 0 for each tapering vessel. Further details on the flow boundary conditions may be obtained from relevant published work (Mynard and Nithiarasu 2008). At the extremities, we assume that the incoming flow is in thermal equilibrium with the surrounding tissue nodes. When the flow is outgoing from the system, the temperature at the down stream point is assigned by characteristic variable extrapolation. As tapering vessels used at the exit boundaries represent microcircualtion, adiabatic conditions are assumed within the these vessels(no heat exchange through convection occurs). A robust modelling requires consideration of branching points such as bifurcations or discontinuities in geometrical and material properties. The works of Mynard and Nithiarasu (2008) and Coccarelli and Nithiarasu (2015) are adopted in the present work.

#### Solid tissues

For the solid tissue representation, we follow the work of  Fiala (1999). The body consists of 14 multilayered cylindrical elements representing head, neck, shoulders, thorax, abdomen, arms, forearms, thighs, and legs (details are reported in Table 1). The segments representing shoulders, legs, thighs, arms, and forearms are constituted by four layers of materials with different properties; from inside to outside the cylinder consists of bone, muscle, fat tissue, and skin layers. In the head, thorax and abdomen segments, inner organs brain, lung and viscera respectively are also included. We notice that some geometrical parameters differ because cylinders have been adapted to the arterial tree.

We note that, in contrast to Fiala (1999), we model head as a cylinder and not like a sphere; thus, the layer radii are resized keeping the head volume constant.

#### Coupling blood with solid systems

The coupling between the blood vessels and surrounding tissue is critical to obtain sensible results. As mentioned previously a one-dimensional bioheat transfer model along the radial direction of body segment is used. The blood vessels are embedded into these segments as shown in Fig. 1. This approach is considerably more advanced than the common assumption of a single core node, which implies that in each cylinder all type of convection losses are depending only on a scalar value. Furthermore, such a model is a good compromise between computational cost and accuracy (Charny et al. 1990).

The artery locations within the solid body are estimated from Uflacker (2006). As reported previously, the heart represents the inlet to the fluid system and is not part of any cylindrical segment. The large arteries proposed in Low et al. (2012) are subdivided into three categories of heart region, central and transversal vessels. As arteries in the heart region are not included in the tissue discretization, no heat transfer with solid tissues occurs; the only exception is represented by the inlet flow node (which is isothermal with surrounding tissues). Each and every central artery is assumed to coincide with the axis of one or more cylindrical segments, while transversal vessels cross transversely one or more cylinders. The arrangement of these vessels within the cylindrical segment is shown in Fig. 1, where central arteries are depicted as chained lines while transversal ones are represented by dashed lines. Figure 2 shows a typical section of the cylindrical segment with embedded central and transversal arteries. It should also be noted that while the geometrical and mechanical properties of elastic vessel may be allowed to change along the longitudinal coordinate, the solid tissue properties along the axial direction of the cylindrical segment are fixed. In addition, cylindrical segments are not considered deformable. The geometrical, thermophysical, and basal physiological properties of tissue materials and the body features are adopted from Fiala (1999). The inner wall heat transfer coefficient is set up following (Shitzer and Eberhart 1985) (Nusselt number is assumed be equal to 4).

Figure 2 clearly shows the spatial discretization adapted in the present study. The body is assumed to be axisymmetric, and the nodes of the central arteries are linked to the first node of the surrounding tissue layers as shown via a convective boundary condition. For every node along the central artery, a matching radial set of nodes are introduced into the surrounding solid tissues. Each central vessel node is therefore identified by two coordinates, a longitudinal and radial coordinate. As shown, the transversal vessels are embedded into the cylindrical segment and at the intersecting point of tissue mesh and transversal artery, a flow node is introduced that coincides with the solid node (see Fig. 2).

The temperature calculation at fluid–solid interface nodes includes the following steps. The temperature transported through the systemic circulation network forms the basis for the boundary condition to Eq. (7). The fluid inlet node (first node of seg. 1) is assumed to be in thermal equilibrium with a tissue node located in the middle of the thorax, having radial coordinate equal to 8 cm. The nodal temperatures of the central arteries provide the wall temperature for the convective boundary condition between the blood and arterial wall (first tissue node). Where a transversal vessel node coincides with the tissue node, a volumetric source term is explicitly evaluated based on the expected convection contribution and added to the discrete heat conduction equation of the tissue (increasing the term $q_m$). In cylinders representing head, neck, legs, and forearms there are no central arteries. Thus, along the axis of these cylindrical segments, an adiabatic condition is adopted.

The perfusion in solid tissue segments is modelled through the perfusion coefficients (see Eq. 7). The temperature difference in the perfusion term is calculated as the difference between the section average blood (mean between all vessels crossing the section) and tissue temperatures. Equation (7) is applied to all tissue nodes by setting the appropriate material constants $k_t$, $\rho _t$, and $c_{t}$, $q_m$ and $\phi$. It should be noted that $q_m$ and $\phi$ are variables regulated by the thermoregulatory system (for further details see Sect. 2.4). In the present study, the tissue temperatures are computed after the evaluation of blood temperatures every time step. All the components of $q_m$ are evaluated before computing the tissue temperature at each time step.

The respiration losses are incorporated by considering a negative volumetric heat source $q_\mathrm{bre}$ at all lung nodes. To estimate such losses the following formulation has been used (Smith 1991)

17 $$\begin{aligned} q_\mathrm{bre}= & {} \frac{1}{V_\mathrm{lung}}[0.0014~Q_{m,\mathrm{glob}}~(34-T_\mathrm{out})\nonumber \\&+\,0.0173~Q_{m,\mathrm{glob}}~(5.87-P_{\mathrm{out}})]~(W/{cm}^3) \end{aligned}$$

where $Q_{m,\mathrm{glob}}$ is the global metabolic heat generation rate, $V_\mathrm{lung}$ is the lung volume (respectively 58.2 $\mathrm{W}/\mathrm{m}^2$ and 5631.41 $\mathrm{cm}^3$) and $P_\mathrm{out}$ is the ambient water vapour pressure. Further details may be found in Smith (1991).

#### Heat exchanged with the environment

The body exchanges heat with the environment through the skin and breathing. The skin is represented by the outer most part of the cylindrical segment. The flux exchanged between the skin layer and outside environment $q_\mathrm{skin}$ is the sum of the convection ($q_\mathrm{con,out}$), radiation ($q_\mathrm{rad}$) and evaporation ($q_\mathrm{eva}$) losses. The Neumann boundary condition used in the present study is

18 $$\begin{aligned} -k_t A_\mathrm{out} \frac{\partial T_s}{\partial r}|_{r_\mathrm{out}}=q_\mathrm{con,out}+q_\mathrm{rad}+q_\mathrm{eva} \end{aligned}$$

For the evaluation of $q_\mathrm{con,out}$ and $q_\mathrm{rad}$ the methodology proposed by Fiala (1999) is followed. The convective heat transfer between skin node and the external environment may be evaluated with the following expression.

19 $$\begin{aligned} q_\mathrm{con,out}=h_\mathrm{con,out}(T_t(r_\mathrm{out})-T_\mathrm{out}) \end{aligned}$$

where $h_\mathrm{out,con}$ is the convection heat transfer coefficient and it is a function of the node location in the body, the air velocity and the temperature difference between the outer surface and environment. For the radiative exchange the evaluation of the mean temperature of the surrounding surfaces ($T_{\mathrm{sur},m}$) is necessary before applying

20 $$\begin{aligned} q_\mathrm{rad}=h_\mathrm{rad}(T_t(r_\mathrm{out})-T_{\mathrm{sur},m}) \end{aligned}$$

where $h_\mathrm{rad}$ is the radiative heat transfer coefficient depending on the temperatures, the emission coefficients and the view factors of the surrounding surfaces considered. As sweating (evaporation) is part of the thermoregulatory system, it is discussed in Sect. 2.4 below.

### Control system

Studies show that a state of thermoneutrality exists when the core and mean skin temperatures of the body are respectively 36.8 and $33.7\,^\circ \hbox {C}$ (Holopainen 2012). When an imbalance in energy exchange between the body and environment occurs, the thermoregulatory system is activated to maintain the body homeostasis. The core body temperature is controlled by the thermoregulatory system consisting of thermoreceptors and hypothalamus. Three control mechanisms, shivering (lower skin temperatures), sweating (higher skin temperatures), and vasomotion (flow control), are considered here. We define $T_\mathrm{core}$ as the mean temperature between the first layer inner nodes of head, neck, thorax and abdomen, while $T_\mathrm{skin}$ is the average value on the skin surface. With such integral variables, we can evaluate the shivering and vasomotion contributions (Smith 1991). The shivering heat per unit volume, $q_\mathrm{shiv}$, may then be obtained by dividing the total segmental heat production by muscle volume. In the present study, the basal and vasomotor blood flows are taken from Smith (1991). The corresponding perfusion rate $\phi$ is evaluated by dividing the flow rate by the skin mass of the segment considered. The total evaporative heat loss $q_\mathrm{swe}$ is computed following (Refrigerating American Society of Heating and Air-Conditioning Engineers 1993). For such calculations we assume that the vapour pressure on skin is equal to that of saturated water vapour at skin temperature Fanger (1970), while the evaporative heat transfer coefficient ($h_\mathrm{swe}$) is taken from  Kerslake (1972). We include also the clothing model proposed in Holopainen (2012).

**Table 2:** Fluid parameters and properties used in the simulations

Density of fluid, $\rho$ $(\mathrm{g}/\mathrm{cm}^3)$	1.060
Viscosity of fluid, $\mu$ (poise)	0.035
Thermal conductivity of fluid, *k* ($\mathrm{W}/\mathrm{cm}^\circ \hbox {C}$)	0.050
Specific heat of fluid, $c_p$ ($\mathrm{J}/\mathrm{g}^\circ \hbox {C}$)	3.900

**Table 3:** Solid properties used in the simulations

Tissue	$c_t$ (J/g K)	$q_{m,0}$ $(\mathrm{W}/{\mathrm{cm}}^3)$	$\rho _t$ $(\mathrm{g}/{\mathrm{cm}^3})$	$k_t$ $(\mathrm{W}/\mathrm{cm} \mathrm{K})$	$\phi$ $(1/\mathrm{s})$
Brain	3.850	0.013400	1.080	0.0049	0.011320
Lung	3.718	0.000600	0.550	0.0028	0.004310
Viscera	3.697	0.004100	1.000	0.0053	0.000500
Bone	1.700	0.000000	1.375	0.0075	0.000000
Muscle	3.700	0.000727	1.085	0.0042	0.000538
Fat	2.300	0.000003	0.850	0.0016	0.000004
Skin	3.680	0.001096	1.085	0.0047	*Variable*

For the cutaneous perfusion, we adopted a specific coefficient for each cylinder [more details can be found in Holopainen (2012)]

## Results and discussion

Although the model proposed in the present work is new and comprehensive, different components of the model have undergone extensive testing in the past. The systemic circulation model, for example, has been extensively used in different studies and compared against experimental flow and pressure measurements (Mynard and Nithiarasu 2008; Low et al. 2012). The temperature transport in the one-dimensional flexible pipes has been studied in detail recently (Coccarelli and Nithiarasu 2015). Thus, the focus of the results in the present study is the bioheat transfer within a human body for various governing parameters. We consider a bare body and thus $R_\mathrm{swe,cl}$ and $f_\mathrm{cl}$ are set respectively equal to 0 and 1 (see “Appendix”). For the cases considered, we assume the same radiative parameters presented in Fiala (1999), while air velocity ($v_\mathrm{air}$) is set equal to 4 m/min. The initial temperature at all nodes are set at $36.8\,^\circ \hbox {C}$ in order to reflect an initial thermo neutral condition. The fluid properties and outside conditions used in the study are listed in Tables 2 and 3.

**Fig. 3:**
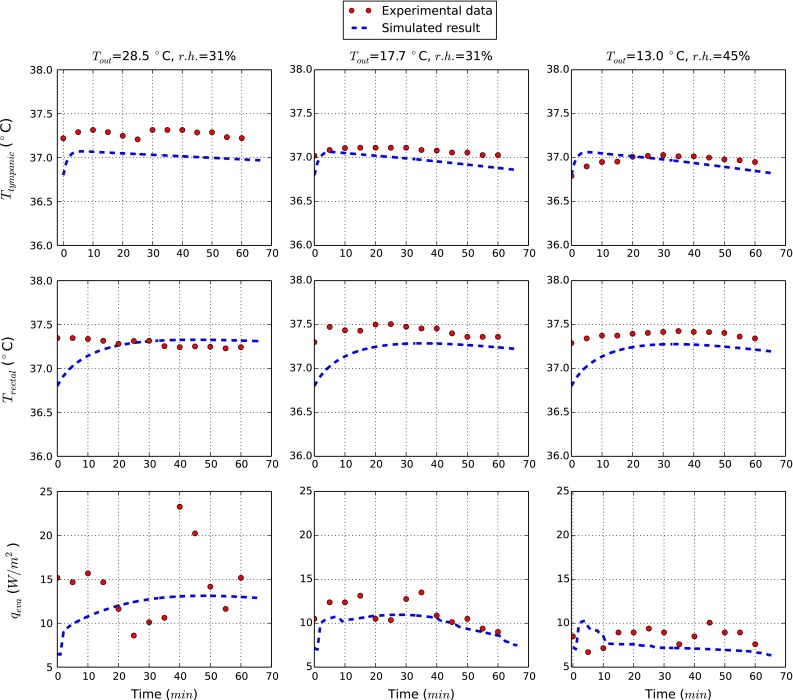
Tympanic and rectal temperatures for various external conditions

In the following subsection, a comparison of the current model for various atmospheric conditions against measurement is provided. This is followed by an investigation on the contribution of inner convection to the body thermal balance and finally the thermoregulatory response of the body in a cold environment is quantified.

### Comparison against measurements

At first a validation of the model with experimental data is presented. For doing this the relevant works by Stolwijk and Hardy (1966), and Hardy and Stolwijk (1966) are used. In these studies volunteers have undergone to various environmental conditions. Temperatures were recorded for the tympanic and rectal regions and also the evaporative losses (sweating and breathing latent losses) were evaluated. In order to test systematically the current model, we simulate the body response for three different external exposures. The considered conditions are ($T_\mathrm{ext}=28.5\,^\circ \hbox {C} - r.h.=31\,\%$), ($T_\mathrm{ext}=17.7\,^\circ \hbox {C} - r.h.=31\,\%$) and ($T_\mathrm{ext}=13.0\,^\circ \hbox {C} - r.h.=45\,\%$). For the rectal temperature calculation, we use the tissue node at an axial distance of 22 cm from the top of abdominal cylinder and at a radius of $r=3.5$ cm. The tympanic site is assumed to be at a distance of 12 cm from the bottom of the head cylinder and at $r=5.0$ cm. The evaporative losses are evaluated by summing the contributions of each cylinder section and then dividing by the total skin surface. We note that the initial temperature field imposed slightly differs from the one of a body under thermoneutral conditions. However, after a long transient all results have to converge to the same value range.

In Fig. 3 the time evolutions of tympanic, rectal temperatures and evaporative losses are reported. For all exposure conditions considered, the simulated results match very well the experimental data. It is possible to see that the temperature errors decrease significantly with the time. At quasi-steady state, the largest difference in temperature is less than $0.25\,^\circ \hbox {C}$. The accuracy of the evaporative losses calculated is difficult to evaluate as the experimental data is widely scattered.

**Fig. 4:**
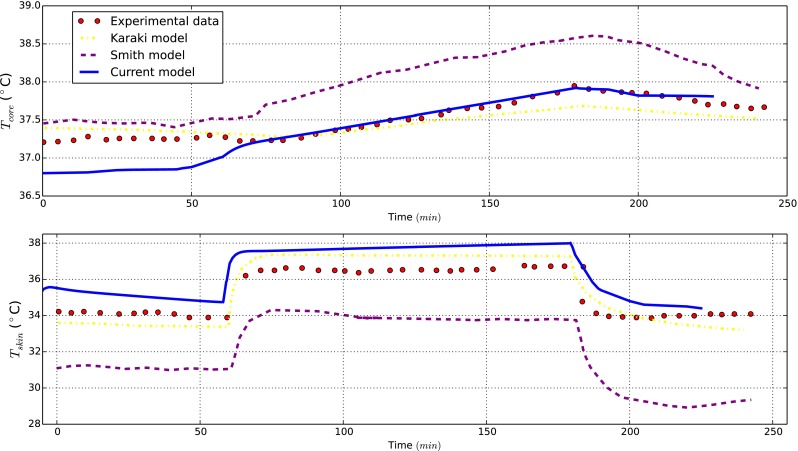
Benchmark case for naked body under hot exposure

Next, we report the thermal body response under controlled external conditions providing comparisons with experimental measurements and other numerical models. Specifically, the model is tested under exposure to heat for 1h at ($28.1\,^\circ \hbox {C},43\,\% \hbox { R.H.}$), 2 h at ($47.8\,^\circ \hbox {C},27\,\% \hbox { R.H.}$) and 1h at ($28.3\,^\circ \hbox {C},44\,\% \hbox { R.H.}$). Findings for these simulated conditions are compared with experimental data (Hardy and Stolwijk 1966) and solutions provided by “Smith” and “Karaki” models, respectively, presented in Smith (1991), Karaki et al. (2013). The core and mean skin temperature behaviours in time are shown in Fig. 4. Our results are inline with the expectations. As seen the mean skin temperature curve rises suddenly as the step change occurs, but then it remains within an acceptable range of temperatures.

**Fig. 5:**
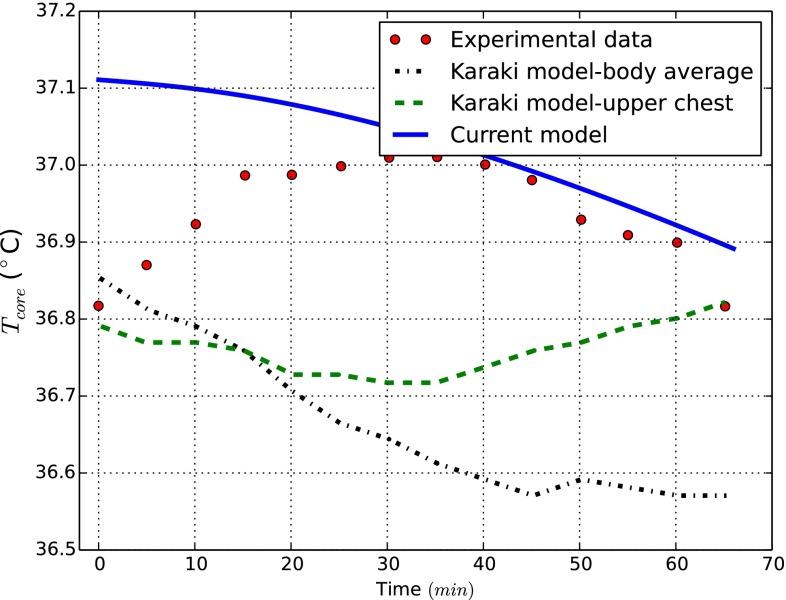
Benchmark case for naked body under cold exposure

The model is also tested when the naked body is exposed to cold conditions. The core temperature prediction is compared against the findings reported in one of the most recent work (Karaki et al. 2013). Here the body is exposed to ($13\,^\circ \hbox {C},45\,\%\hbox { R.H.}$) for 65 min. The results are reported in Fig. 5. As seen, a good agreement with experimental data is evident.

### Role of inner convection

To understand the temperature changes in blood, four representative arteries, abdominal aorta II (seg. 43, abdomen), left external carotid (seg. 25, head), right external iliac (seg. 58, right thigh), and right radial (seg. 16, right arm), are selected (for more detail about artery labelling see Low et al. 2012). The temperatures at these locations are recorded once a quasi-steady state is reached. The tissue temperature distributions are recorded for the sections corresponding to the nodes selected in the mentioned arteries (abdomen, head, thigh, and arm).

Since the flow is pulsatile in nature (Low et al. 2012), pulsatility of temperature is also anticipated. In addition, the wave nature of the flow leads to reflected temperature waves. Although a number of different parameters such as elastic properties of the vessels can be tested using the proposed model, all the material properties, heart rate and flow boundary conditions at the extremities are fixed to produce an understanding of a normal human body behaviour. Note that describing bioheat transfer in a body subjected to some disease states or extreme environmental conditions needs parameter changes.

**Fig. 6:**
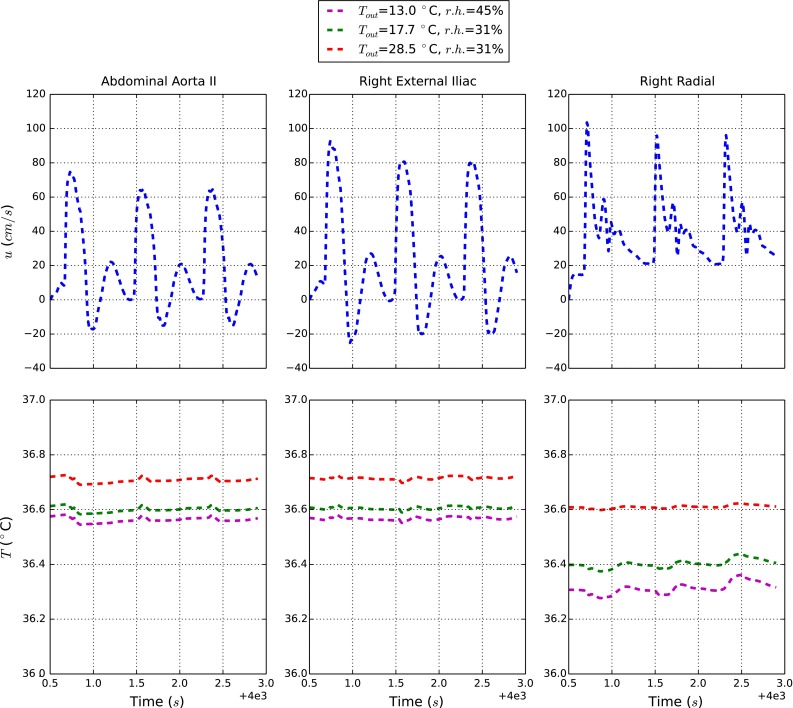
Blood velocity and temperature along the arterial tree for various external conditions

Figure 6 shows the blood temperature at three selected monitoring points in the systemic circulation. As anticipated, the temperature follows a mild periodic pattern inline with the velocity changes. As seen the frequency and amplitude of oscillations differ for different environmental conditions. In general the amplitude of the temperature waves is low, and thus no dramatic local change in temperature is possible. The blood temperature is mildly influenced by the atmospheric temperature in the core part of the body. The pronounced effect in the radial artery is due to the smaller dimensions of the forearm and to the absence of any metabolic active tissue.

**Fig. 7:**
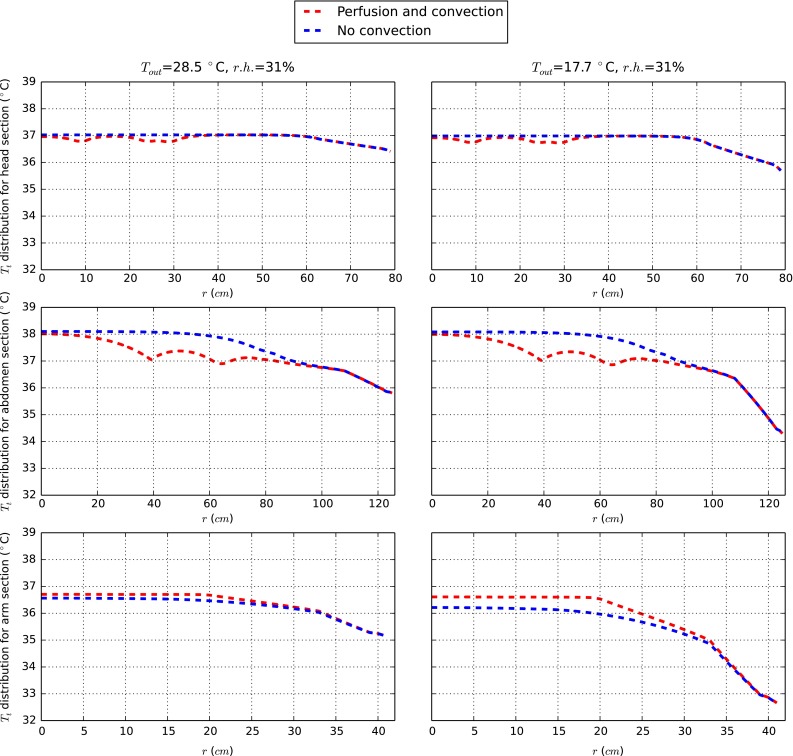
Tissue temperatures for two different modelling approaches at *t* = 33.0 min

In order to evaluate the effect of heat convection on tissues, the results obtained from the proposed model are compared to the approach used in other reference works (Fiala 1999; Tanabe et al. 2002) where heat conduction is exclusively used to model heat transfer occurring between blood and tissue system. Figure 7 shows the temperature distribution with and without heat convection and perfusion in arteries. As seen a local temperature variation of more than $1.0\,^\circ \hbox {C}$ is observed in tissues in the abdominal area. Although this variation decays as we approach the skin layer, this finding is important for further investigation. In the abdomen and head, convection involves a smaller average tissue temperature compared to the case without convection. The situation in the arm instead is the opposite. This can suggest that, with convection, a more uniform energy redistribution is enforced. It can therefore be reasonably concluded that flow and convection heat transfer play an important regulatory role that may be further enhanced in abnormal conditions such as high blood pressure and stiffer arteries.

### Influence of thermoregulation

It is often difficult to evaluate the effect of thermoregulation as this is highly coupled with different external parameters. Thus, in this section an example is provided to demonstrate the effect of thermoregulation when the body is subjected to a cold exposure. For doing this we consider also a case in which all control mechanisms (shivering, cutaneous vasomotion and sweating) are shut down. All other parameters are assumed to be the same as the previous subsections.

**Fig. 8:**
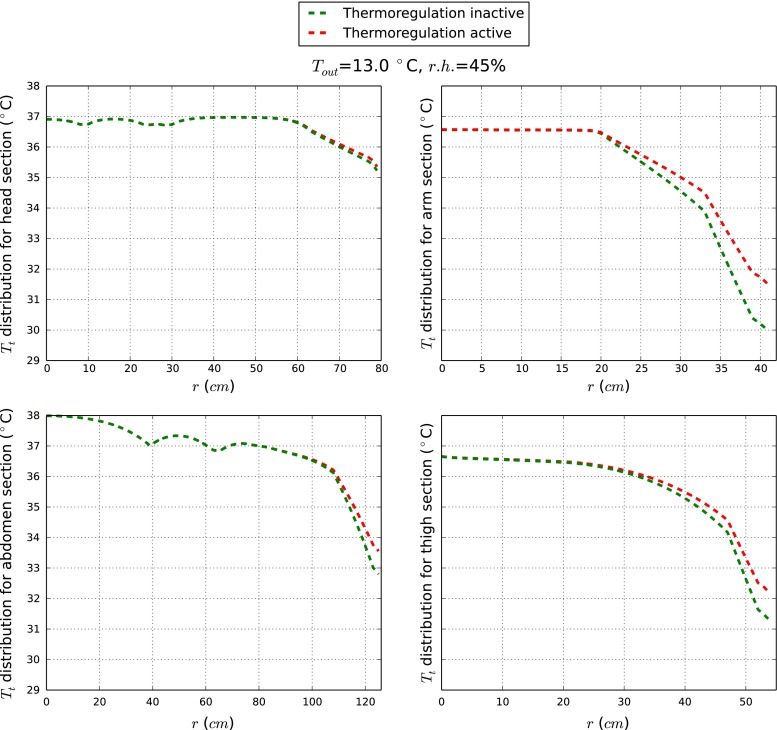
Thermoregulation effects on tissue temperatures at *t* = 33.0 min

Figure 8 highlights the influence of the thermoregulatory system in all the four regions considered. While the core temperature remains approximately the same, the temperature at the periphery has dropped without thermoregulation. Most consistent temperature variations occur in the peripheral body cylinders (arm and thigh). The reduction after 33.0 min is as high as $1\,^\circ \hbox {C}$. The shivering effect is not included as the core temperature needed to trigger shivering has not been reached. For a longer time or more extreme external conditions, such profiles could change significantly.

## Conclusions

A novel, next-generation bioheat model for the human body has been developed and tested. The systemic circulation embedded human body model is more comprehensive than existing models. Further improvements are nevertheless possible by including more arteries and veins. The proposed model in its present form can test various parameters including artery stiffness, blood pressure, various dimensions, tissue properties, surrounding conditions and many more. The results produced very clearly highlight the effect of arterial heat convection on the surrounding tissues. The heat convection and perfusion enhances the energy exchange between the blood and surrounding tissues. As expected surrounding temperature changes significantly affect the skin temperature; however, the control system limits the rapid variation of temperature whenever outside temperature is far from thermally neutral conditions.

There are numerous potential applications of the proposed model, such as better understanding of hyperthermia/hypothermia and the detailed study of resulting temperature transport and distribution. Besides these applications, the proposed model can study the influence of disease conditions such as hypertension and arterial dysfunction and also ageing on energy exchange. The model can also be used to study changing environments as a condition for enhanced quality of life.

## Figures and Tables

**Fig. 9:**
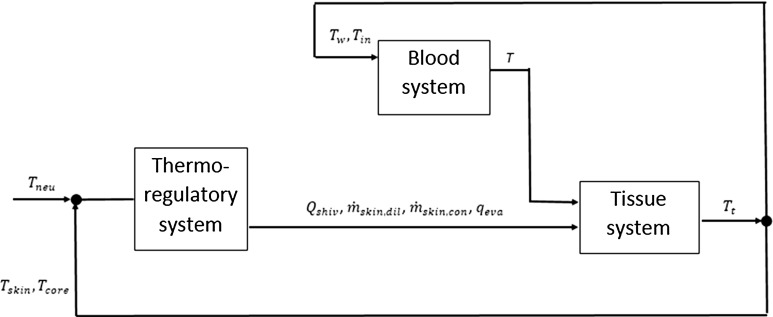
Global system

## Appendix

### System interconnections and solution procedure

Here we describe how all the subsystems are interconnected and coupled (see Fig. 9). The thermoregulatory response is evaluated by knowing $T_\mathrm{core}$ and $T_\mathrm{skin}$ of the previous time step and comparing them with thermoneutrality reference values. Such control system is able to modify tissue balance through shivering heat source, increment or decrement of skin perfused flow, sweating losses. As blood variables are evaluated in an explicit way, we use $T_t$ of the previous time step for prescribing interacting wall and fluid inlet temperatures. Once blood system output are calculated, tissue temperatures are calculated before starting a new cycle.

The calculation procedure for evaluating temperatures of the global system at each time step is carried out as follows:

1. $T^{n+1}$ is calculated explicitly by means of third equation of (3) using $T_w^n$;
2. At the extremities of fluid network $T^{n+1}$ are assigned equal to $T_t^n$ of the interacting tissue nodes;
3. $T_\mathrm{core}$ and $T_\mathrm{skin}$ are derived from $T_t^n$ field;
4. Convection from transversal vessels, breathing, sweating and shivering contributions are calculated using $T^{n+1}$ and $T_t^{n}$;
5. $T_t^{n+1}$ is computed implicitly with Eq. (7).

### Thermoregulatory system equations

Here the most relevant equations for modelling regulation processes are reported.

#### Shivering

From Smith (1991) it is assumed that the shivering temperature, $T_\mathrm{shiv}$, is a function of the core temperature, i.e.,

21 $$\begin{aligned} {\left\{ \begin{array}{ll} T_\mathrm{shiv}=35.5^\circ \mathrm{C}~~if~~T_\mathrm{core}< 35.8~{^\circ \mathrm{C}} \\ T_\mathrm{shiv}=-10222+570.9~T_\mathrm{core}-7.9455~T_\mathrm{core}^2~({^\circ \mathrm{C}})\\ ~~if~~35.8~{^\circ \mathrm{C}}\le T_\mathrm{core} \le 37.1~{^\circ \mathrm{C}} \end{array}\right. } \end{aligned}$$

It should be noted that for $T_\mathrm{core}$ greater than 37.1 $^\circ \mathrm{C}$, shivering does not occur. The maximum increase in total metabolic heat generation caused by shivering ($Q_\mathrm{shiv,max}$) may be written as

22 $$\begin{aligned} Q_\mathrm{shiv,max}= & {} \frac{1}{3600}(-1.1861~10^9+\,6.552~10^7~T_\mathrm{core} \nonumber \\&- 9.0418~10^5~T_\mathrm{core}^2)~(W) \end{aligned}$$

The shivering metabolic heat generation $Q_\mathrm{shiv}$ may now be calculated as

23 $$\begin{aligned} Q_\mathrm{shiv}= & {} Q_\mathrm{shiv,max}\left[ 1-\left( \frac{T_\mathrm{skin}-20}{T_\mathrm{shiv}-20}\right) ^2\right] ~(W)\nonumber \\&~~\mathrm{if}~~(40-T_\mathrm{shiv}) \le T_\mathrm{skin} \le T_\mathrm{shiv}~({^\circ \mathrm{C}}) \end{aligned}$$

#### Vasodilation and vasoconstriction

Vasodilation and vasoconstriction respectively increase and decrease arterial flow in the skin layers. To model these processes, we follow the method proposed in Smith (1991). At thermoneutrality condition, flow assumes a basal value ($\dot{m}_\mathrm{skin,bas}$). Whenever core temperature increases over its neutral value, vasodilation occurs. When the core temperature reaches $37.2\,^\circ \hbox {C}$, the maximum flow in the skin layer is recorded ($\dot{m}_\mathrm{skin,max}$). Between the core temperatures of 36.8 and 37.2 $^\circ \hbox {C}$, the skin blood flow follows the core temperature linearly. As mean skin temperature falls below its neutral value of 33.7 $^\circ \hbox {C}$, vasoconstriction occurs. The state of maximum vasoconstriction is recorded for a mean skin temperature equal to 10.7 $^\circ \hbox {C}$ (Smith 1991). At this temperature the skin blood flow assumes a minimum value ($\dot{m}_\mathrm{skin,min}$). Between skin temperatures of 33.7 and $10.7\,^\circ \hbox {C}$ the skin blood flow is assumed to vary linearly with temperature.

The evaluation of vasodilation and vasoconstriction flows ($\dot{m}_\mathrm{skin,dil}$ and $\dot{m}_\mathrm{skin,con}$) for a body segment may be calculated via the following expressions:

24 $$\begin{aligned} {\left\{ \begin{array}{ll} \dot{m}_\mathrm{skin,dil}=\dot{m}_\mathrm{skin,bas}~(\mathrm{kg}/\mathrm{s})~~\mathrm{if}~~T_\mathrm{core}< 36.8 ^\circ \mathrm{C} \\ \dot{m}_\mathrm{skin,dil}=\frac{T_\mathrm{core}-36.8}{37.2{-}36.8}(\dot{m}_\mathrm{skin,max}-\dot{m}_\mathrm{skin,bas}) \\ \quad +\,\dot{m}_\mathrm{skin,bas}~(\mathrm{kg}/\mathrm{s}) ~~\mathrm{if}~~36.8 ^\circ \mathrm{C}\le T_\mathrm{core} \le 37.2 ^\circ \mathrm{C} \\ \dot{m}_\mathrm{skin,dil}=\dot{m}_\mathrm{skin,max}~(kg/s)~~\mathrm{if}~~T_\mathrm{core} > 37.2 ^\circ \mathrm{C} \end{array}\right. } \end{aligned}$$

and

25 $$\begin{aligned} {\left\{ \begin{array}{ll} \dot{m}_\mathrm{skin,con}=\dot{m}_\mathrm{skin,min}~(\mathrm{kg}/\mathrm{s})~~\mathrm{if}~~T_\mathrm{skin}< 27.8 ^\circ \mathrm{C} \\ \dot{m}_\mathrm{skin,con}=\frac{T_\mathrm{skin}-27.8}{33.7{-}27.8}(\dot{m}_\mathrm{skin,bas}-\dot{m}_\mathrm{skin,min}) \\ \quad +\,\dot{m}_\mathrm{skin,min}~(\mathrm{kg}/\mathrm{s}) ~~\mathrm{if}~~27.8 ^\circ \mathrm{C} \le T_\mathrm{skin} \le 33.7 ^\circ \mathrm{C} \\ \dot{m}_\mathrm{skin,con}=\dot{m}_\mathrm{skin,bas}~(\mathrm{kg}/\mathrm{s})~~\mathrm{if}~~T_\mathrm{skin} > 33.7 ^\circ \mathrm{C} \end{array}\right. } \end{aligned}$$

#### Sweating

The sweating threshold $T_\mathrm{swe}$ is approximated as a function of mean skin temperature as (Refrigerating American Society of Heating and Air-Conditioning Engineers 1993):

26 $$\begin{aligned} {\left\{ \begin{array}{ll} T_\mathrm{swe}=42.084-0.15833~T_\mathrm{skin}~({^\circ \mathrm{C}}) \\ \quad \mathrm{if}~~T_\mathrm{skin}\le 33.0~^\circ \mathrm{C}\\ T_\mathrm{swe}=36.85~^\circ \mathrm{C}~~if~~ T_\mathrm{skin} > 33.0~^\circ \mathrm{C} \end{array}\right. } \end{aligned}$$

The sweat rate $\dot{m}_\mathrm{swe}$ may now be evaluated as

27 $$\begin{aligned} \dot{m}_\mathrm{swe}= \frac{45.8+739.4(T_\mathrm{core}-T_\mathrm{swe})}{3.6~10^6}~(\mathrm{kg}/\mathrm{s}) ~~\mathrm{if}~~ T_\mathrm{core} > T_\mathrm{swe} \end{aligned}$$

The relative skin wetness *w* is given as

28 $$\begin{aligned} w=0.06+\frac{\dot{m}_\mathrm{swe}(1-0.06)}{0.000193} \end{aligned}$$

The total evaporative heat loss $q_\mathrm{swe}$ may now be written as (Refrigerating American Society of Heating and Air-Conditioning Engineers 1993)

29 $$\begin{aligned} q_\mathrm{swe}=\frac{w(P_\mathrm{skin}-P_\mathrm{out})}{R_\mathrm{swe,cl}+\frac{1}{f_\mathrm{cl}h_\mathrm{swe}}}~(W/m^2) \end{aligned}$$

where $P_\mathrm{skin}$ is water vapour pressure on skin, $R_\mathrm{swe,cl}$ is the evaporative heat transfer resistance of the clothing layer, $f_\mathrm{cl}$ is the clothing area factor (the surface of the clothed body divided by the area of the bare body), and $h_\mathrm{swe}$ is the evaporative heat transfer coefficient.
